# Lead, Cadmium, and Arsenic in Edible Tissues of Guinea Pigs Raised in the Central Andes of Peru: Potential Human Health Risk?

**DOI:** 10.3390/vetsci12040292

**Published:** 2025-03-21

**Authors:** Doris Chirinos-Peinado, Jorge Castro-Bedriñana, Fiorela Rivera-Parco, Elva Ríos-Ríos

**Affiliations:** 1 Food and Nutritional Security Research Group, Universidad Nacional del Centro del Perú, Huancayo 12000, Peru; dchirinos@uncp.edu.pe; 2 Faculty of Zootechnics, Universidad Nacional del Centro del Perú, Huancayo 12000, Peru; fioana2023@gmail.com; 3 Faculty of Sciences, Universidad Nacional Agraria La Molina, Lima 15024, Peru; erios@lamolina.edu.pe

**Keywords:** toxic metals, toxic metalloids, food contamination, health risk, target hazard quotient, hazard index, food toxicology, environmental exposure, food security, Central Andes

## Abstract

Toxic metals and metalloids food contamination is a source of health risks; this study evaluated lead (Pb), cadmium (Cd), and arsenic (AS) contamination in the meat, liver, kidneys, heart, and lungs of guinea pigs raised on a farm near a mini mineral concentrator and with pastures irrigated with wastewater in the Central Andes of Peru. The risk of consuming these elements was estimated in the Peruvian population aged 2 to 85 years, whose consumption is 660 g/per capita/year. The quantification of Pb, Cd, and As was carried out via Inductively Coupled Plasma Atomic Emission Spectrometry. The heart had 3.3, 4.3, 7.3, and 81 times more Pb than the liver, lung, kidneys, and meat. The liver had 1.02, 2.22, 9.15, and 722.5 times more Cd than the kidneys, heart, lungs, and meat, respectively. The kidneys had 1.16 and 1.72 times more As than the liver and heart, respectively. The Target Hazard Quotient (TQH) and Hazard Index (HI) for Pb, Cd, and As, were <1 in the entire population, and there was no risk from their consumption. The cancer risk values found in the study are within the tolerated ranges. The findings expand the scientific basis for the safe and innocuous production of guinea pigs in the Central Andes of Peru and provide evidence to establish limits on water, soil, pastures, and agri-food products.

## Simple Summary

This pioneering study analyzed, for the first time, the presence of toxic metals and metalloid content in guinea pig meat and its relationship with its chemical composition in the context of exposure to mining contamination and wastewater irrigation in the Central Andes of Peru. The findings indicate that although lead (Pb) accumulated in greater quantities in the heart, cadmium (Cd) was higher in the liver, and arsenic (As) was higher in the kidneys, with accumulation observed in specific organs, the levels in the meat remained within permissible limits and did not pose a risk to the health of the Peruvian population between 2 and 85 years of age. These results are critical for the regulation of toxic metals and metalloid contamination in food production in the Central Andes of Peru, contributing to food security and public health in the region.

## 1. Introduction

Potentially toxic elements, such as cadmium (Cd), lead (Pb), nickel (Ni), chromium (Cr), mercury (Hg), and arsenic (As), pose an environmental and health risk due to their high density (>5 g/cm^3^), toxicity, and ability to bioaccumulate in the food chain. Their presence, from both natural and industrial sources, can seriously affect living organisms, making their monitoring and control a global priority [1].

There is growing concern about food contamination with toxic metals and metalloids, particularly in rural areas of the Peruvian Andes, where mining activity and the use of wastewater for irrigation represent significant sources of environmental exposure [2]. Recent studies indicate the presence of Pb and Cd in soil and pastures, and their concentrations are increasing due to mining/metallurgical activities [3], the use of phosphorus agrochemicals containing cadmium [4], and the use of wastewater for irrigation [1], with the presence of As reported in milk from cows raised in the Central Andes [5]. These metals emitted in the form of fine particles in smoke and vapor are transported many kilometers and deposited mainly in the soil and water, incorporating themselves into the production chain and bioaccumulating in the food consumed by humans [6,7,8,9,10], including dairy products and meats, such as that of guinea pigs.

In Peru, the country with the largest production of guinea pigs (*Cavia porcellus*) worldwide, and other developing countries, in recent years, the breeding of these small meat rodents has experienced sustained growth, becoming an important source of animal protein for the local population and a source of income for Andean populations, contributing to food security in these populations [11,12,13]. This trend is due to the nutritional qualities of guinea pig meat, which is rich in proteins and essential minerals, such as calcium, iron, and zinc, and low in saturated fat [12].

Genetic improvements have been implemented to optimize growth, performance, and reproduction [14] but contamination with toxic metals and metalloids in water, soil, and plants poses risks to carcass safety and human health.

Pb, Cd, and As are known for their toxic and cumulative effects on animal and human tissues, and their chronic exposure can trigger serious health problems, including neurological, renal, and carcinogenic effects [15,16]. The concentration of these metals in biological and clinical samples from cancer patients was higher than that of healthy individuals [17]. In this context, the detection and quantification of these toxic metals and metalloids in guinea pig tissues is essential to assess potential risks to consumers and ensure food safety. According to previous studies, animal meat and their organs can represent a significant source of exposure to heavy metals through the food chain [18,19]; in this case, when they are raised in areas close to mineral concentrators and the forage irrigation is with wastewater, it is crucial to understand potential exposure levels and non-carcinogenic risks through guinea pig consumption.

Meat consumption, in general, has been increasing in recent decades [20], which underlines the importance of monitoring the quality of this food. The consumption of guinea pig meat and offal is an integral part of the traditional diet of Andean peoples and is a fundamental and emblematic pillar of Peruvian gastronomy, with a rich, millennia-long history. The carcass is made up of the skinned guinea pig without digestive viscera; it includes the skin, head, liver, kidneys, heart, and lungs, and in Peru, it is consumed by children and older adults. However, there are few studies that explore heavy metal contamination in guinea pig carcasses and the associated risks to human health and food safety [12]. In the province of Huancayo, located in central Peru, where there are several guinea pig farms, a worrying accumulation of heavy metals has been detected in soils and pastures [3] as a result of agricultural and livestock activities, including the excessive use of phosphorus fertilizers, proximity to an old waste dump, and irrigation of pastures with wastewater [21]. These contaminated conditions not only affect the quality of soils and food but also pose a risk to animals raised on these lands, such as guinea pigs, whose edible organs, such as the heart, liver, lungs, and kidneys, can accumulate these toxic metals and metalloids, given their capacity for bioaccumulation [19].

This study’s objective was to determine the level of Pb, Cd, and As in the meat, liver, kidneys, heart, and lung of 4-month-old fattening guinea pigs raised in the Central Andes of Peru impacted by heavy metal contamination, comparing the values obtained with the established maximum permissible limits and evaluating the health risks through the Target Hazard Quotient (THQ) and the Hazard Index (HI) derived from the consumption of guinea pig in the Peruvian population aged 2–85 years.

This analysis is crucial not only to safeguard the health of consumers but also to provide a scientific basis for implementing policies, safer practices in guinea pig breeding, and food control measures in the Andean region, helping to protect the population and promoting safer agricultural and livestock practices.

## 2. Materials and Methods

### 2.1. Ethical Aspects

ARRIVE 2.0 guidelines on experimental design and reporting standards for animal research were followed throughout the research. All animal procedures were approved by the General Research Institute of the National University of Central Peru and Rectoral Resolution No. 1435-R-2023. The number of animals used in this study was considered minimal in order not to jeopardize the study’s objectives. The guinea pigs were euthanized by cervical dislocation prior to slaughter, a procedure that minimizes pain and suffering to the animals.

### 2.2. Study Location

**Figure 1:**
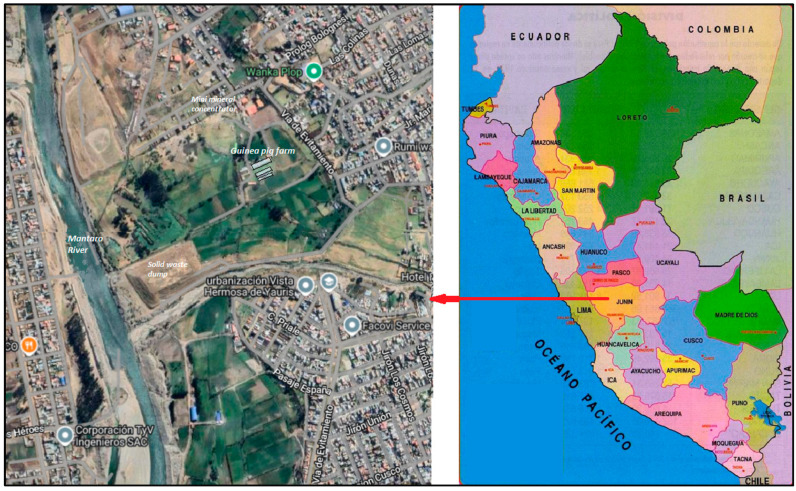
Map showing the location of the guinea pig farm, Huancayo, Peru. Source: Google Maps.

The guinea pigs in this study belong to a representative farm of meat guinea pigs, located in the district of Huancayo, province of Huancayo, on the urban periphery on the right bank of the Mantaro River (Figure 1). The climate of the study area is characterized by two well-defined seasons. The rainy season is from October to March, and the dry season is from April to September. According to Köppen and Geiger, the climate is classified as ET. The average annual temperature and rainfall are 8.7 °C and 1682 mm.

### 2.3. Animals and Breeding System

The guinea pigs in this study were between 4 and 4.5 months old, with a market live weight between 1100 and 1200 kg, adults; they were raised in wooden and wire mesh pools and had access to cultivated pastures and cut alfalfa (*Medicago sativa*) and barley (*Hordeum vulgare*), which were both produced on the same farm. The pastures were irrigated with water from a spring near the Mantaro River and with wastewater without any prior treatment.

In a study conducted on the same farm, the presence of Pb, Cd, and As in the soil was reported at 292 mg/kg, 3.54 mg/kg, and 1.58 mg/kg, respectively. In the grass used in animal feed, the concentrations of these toxic elements were 23.17 mg/kg, 0.25 mg/kg, and 0.06 mg/kg, respectively [5].

### 2.4. Sample Preparation and Metal and Metalloid Determination

#### 2.4.1. Sampling of Guinea Pig Meat and Offal

For this study, from a batch of 100 guinea pigs that finished fattening, a total of 10 guinea pigs were randomly selected from a representative farm in the district and Huancayo province. The guinea pigs were sacrificed and 5 samples were obtained per guinea pig, totaling 50 samples: 10 meat, 10 liver, 10 kidney, 10 heart, and 10 lungs.

To sacrifice the guinea pigs, they are held by their hind legs, hit on the back of the neck to numb them, and then a cut is made in their neck (slit throat), causing bleeding, a good evacuation of blood with a minimum time of 2 min per animal, and thus the death of the animal. Scalding is performed in a container with water maintaining a minimum temperature of 65 °C. Hair removal is performed manually and then it is washed to eviscerate it, removing the red and white viscera and appendages. The carcasses are then aired and weighed, and they are deboned to obtain meat, fat, and skin and to separate the liver, kidneys, heart, and lungs for shipment to the laboratory. The samples are kept at a temperature of −18 °C to maintain the cold chain in expanded polystyrene boxes and dry ice until the samples are digested by microwave [22].

#### 2.4.2. Preparation of Biological Samples for Toxic Metal and Metalloid Analysis

The preparation of meat and offal samples and the quantification of Pb, Cd, and As were carried out in the accredited Laboratory of the National Institute of Agrarian Research of Huancayo.

Biological samples (2 g/fresh samples) were digested using method 3050B [23], which involves repeated additions of nitric acid (HNO_3_) and hydrogen peroxide (H_2_O_2_) of analytical reagent grade (Inorganic Ventures, Christiansburg, VA, USA) for digestion. Deionized water was used to prepare all solutions. Standard solutions (99.99%, ICP grade) of Pb (Lot S2-PB708977), Cd (Lot S2-CD705778), and As (Lot S2-AS766036), corresponding to 1000 mg/kg of each element, were prepared by sequential dilution. Samples were cooled to room temperature to prevent foaming and splashing, and digestion vessels were uncovered in a fume hood. Each solution was filtered into a 100 mL volumetric flask using Whatman filter paper (0.45 µm) to remove any suspended residue. A total of 14 mL of 1% HNO_3_ was added to the solution and diluted to the mark with deionized water. Digestion of the blanks was also performed in parallel with the meat and offal samples with all digestion parameters being the same.

#### 2.4.3. Metal and Metalloid Determination in Guinea Pig Meat and Offal for Human Consumption

Pb, Cd, and As measurements were carried out with MP-AES (Agilent 4100, Agilent Techologies, Inc., Santa Clara, CA, USA), Inductively Coupled Plasma Atomic Emission Spectrometry [24,25], performing a prior optimization, using standard solutions of the three elements to give the maximum signal intensity by adjusting parameters such as wavelength, nebulizer flow, pump speed, and flow current the lamp for each element (Table 1).

Calibration curves for Pb, Cd, and As were plotted using linear regression analysis of the concentrations of the standard solutions versus the emission values. A series of standard solutions were prepared by diluting the intermediate standard solution with deionized water. The same analytical procedure was used for the determination of the elements in digested blank solutions.

The Limit of Detection (LoD) and Limit of Quantification (LoQ) values in mg/L for MP-AES 4210 for Cd were 0.001 and 0.003; for Pb, they were 0.001 and 0.003; and for As, the EPA method 200.7 was used [26].

The analytical method (MP-AES) had good linearity, evidencing its reliability in determining trace levels of Pb, Cd, and As in guinea pig meat and offal. The concentrations of each element were determined from calibration curves plotted for emission versus concentration. Duplicate determinations were performed for each sample and the average results were reported. Blank values were also measured.

#### 2.4.4. Precision and Accuracy

Analytical results must have precision and accuracy, and in our study, the precision of the results was evaluated by the percentage relative standard deviation of the results of three samples (*n* = 3) and triplicate readings per sample, with nine measurements for a given sample. In this study, recovery rates were between 99.5% and 99.9%.

Duplicate samples allowed for determining the precision of the method and calculating the mean and coefficient of variation, which was less than 5%. The precision is measured using standard solutions of each element, determining the relative error, which, in percentage, represents the precision of the method and must be greater than 95%. For these calculations, standard solutions of Pb, Cd, and As, with 155, 150, and 50 mg/kg of sample, were used [27]. At the time of analysis, the corresponding concentrations were 148.14, 152.50, and 49.58 mg/kg, which are values that, when transformed into percentages, indicate that the method complies with the precision parameters.

### 2.5. Maximum Limits of Pb, Cd, and As in Meat and Meat Products

The National Health Commission and the State Market Regulation Administration of the United States Department of Agriculture (USDA) published the National Food Safety Standard for Maximum Levels of Contaminants in Food, GB 2762-2022, which updates the 2017 regulation published as GB 2762-2017, this report includes the limits of Pb, Cd, and As in meat and meat products, including edible viscera of livestock and poultry [28], which are shown in Table 2.

The National Health Commission and the State Market Regulation Administration of the United States Department of Agriculture (USDA) published the National Food Safety Standard. Considering that there is no regulation of maximum limits for guinea pig meat, in this study, we have used the values proposed for livestock and poultry [28], which will give us a good approximation regarding the consumption of guinea pig meat.

### 2.6. Risk Assessment

The Target Hazard Quotient (THQ) is used to assess the potential non-carcinogenic effects associated with long-term exposure to toxic metals and metalloids in food, and the Hazard Index (HI) assesses the chronic risk of multiple heavy metals [9,29], previously proposed by [30].

An exposure assessment was carried out for the Peruvian population aged 2 to 85 years using the average levels of Pb, Cd, and As in the portion of guinea pig meat consumed, which, according to official reports from Peru, is 660 g per capita/year [31], and the body weights of the population aged 2–85 years. This unique national study included 62,600 people aged 2 to 85 years whose data are current, and there is no other study of this magnitude [32].

The THQ and the Hazard Index (HI) for Pb, Cd, and As from the consumption of guinea pig meat toxicologically demonstrate whether the meat produced in the study area is within the levels established by the Codex Alimentarius [33] and whether these toxic metals and metalloids pose risks to human health [34,35,36].

The potential chronic non-carcinogenic risk of heavy metals, expressed as THQ, was calculated as follows [37,38]:

$$THQ=\frac{EF\times D\times DFC\times MC}{RfD\times WB\times TA}$$

The Exposure Frequency (*EF*) and the period of exposure equivalent to longevity (*ED*) for an adult are considered to be 365 days per year and a lifespan of 70 years.

*DFC* is the daily consumption of guinea pigs in kg/day: 0.002.

*MC* is the average concentration of toxic metals and metalloids in milk in µg/kg.

*RfD* is the oral reference dose of each metal [39,40,41].

- Lead: 0.0035 mg/kg of body weight/day
- Cadmium: 0.001 mg/kg of body weight/day
- Arsenic: 0.003 mg/kg of body weight/day

*WB* is the body weight in kg.

*TA* is the average lifespan in days which is 25,550 days (70 × 365).

The HI is used to assess the potential long-term risk to human health when two or more toxic metals and metalloids are involved. It is the sum of the THQs [42,43]. It indicates the likely risk of non-cancer diseases. A value > 1 indicates a potential risk of health effects; if the HI is <1, no adverse health effects are expected [44].

The carcinogenic risk (CR), which is the probability that an individual will develop cancer from oral exposure to an environmental carcinogen level over time, was computed based on the USEPA Human Health Risk Assessment model and calculated using the following equation: RC = IDE × SF, where IDE is the estimated daily intake and SF is the cancer slope factor (SF), which is the factor of metals considered carcinogens.

The applied oral slope factors were as follows: 0.0085 (mg kg/day) for Pb, 15 (mg kg/day) for Cd, and 1.5 (mg kg/day) for As. For risk management purposes, a cancer risk from 1 × 10^−6^ to 1 × 10^−4^ is considered acceptable or tolerable [45].

### 2.7. Data Processing

Data were analyzed using SPSS 23.0 (IBM, Endicott, NY, USA). The results were expressed as mean ± standard deviation (SD). A one-way ANOVA was used to compare the average values of the metals in the different samples of guinea pig meat and edible offal. The maximum limits used for Pb, Cd, and As in guinea pig meat and offal are shown in Table 1. Graphs were also prepared indicating the THQ and HI curves for the toxic metals and metalloids studied in the Peruvian population aged 2–85 years. In order to explain the concentrations of these metals in the different edible organs of the guinea pig, a radial graph was prepared.

## 3. Results

### 3.1. Edible Components of the Guinea Pig Carcass

In an average 800 g carcass, meat represents more than 90% of this portion, with edible offal representing less than 10%, which concentrates different amounts of heavy metals (Table 3).

These results have served to determine the content of these toxic metals and metalloids in the average daily per capita consumption of guinea pig meat, which is 0.0018 kg.

### 3.2. Lead Levels in Meat, Kidneys, Lungs, Heart, and Liver of Guinea Pigs

The highest concentration of Pb in the guinea pig carcass is recorded in the heart, which had 3.3, 4.3, 7.3, and 81 times more than the liver, lung, kidneys, and meat (muscles and skin), respectively. This result shows that the edible offal is the one that concentrates a greater amount of lead, while the meat (muscle mass) of the legs and arms, representing 93.7% of the edible portion, had less Pb than the rest of the components of the edible portion.

The concentration of Pb in meat was below the MPL (0.2 mg/kg), while the concentrations of Pb in heart, liver, lung, and kidneys were 11.12, 3.34, 2.62, and 1.53 times higher than the MPL for cattle and poultry viscera (0.5 mg/kg) (Table 4).

### 3.3. Cadmium Levels in Meat, Kidneys, Lungs, Heart, and Liver of Guinea Pigs

The highest concentration of Cd in guinea pig carcasses was found in the liver and kidneys, followed by the heart, lungs, and meat. The liver had 1.02, 2.22, 9.15, and 722.5 times more than the kidneys, heart, lungs and meat, respectively.

The concentration of Cd in meat was below the MPL (0.1 mg/kg), while in the liver, kidneys, heart, and lungs, the Pb concentration exceeded the MPL for cattle and poultry viscera by 2.89, 1.42, 1.30, and 0.32 times (Table 5).

### 3.4. Arsenic Levels in Meat, Kidneys, Lungs, Heart, and Liver of Guinea Pigs

The highest concentration of As in the guinea pig carcass was recorded in the kidneys, followed by the liver and heart. No As was detected in the meat and lung, but it is possible that concentrations were below the detection limit.

The kidneys had 1.16 and 1.72 times more than the liver and heart. The concentrations of As in the kidneys, liver, and heart exceeded the MPL (0.5 mg/kg) for viscera of cattle and poultry by 3.21, 2.77, and 1.87 times (Table 6).

### 3.5. Comparison of Lead, Cadmium, and Arsenic Concentrations in Guinea Pig Meat and Offal

**Figure 2:**
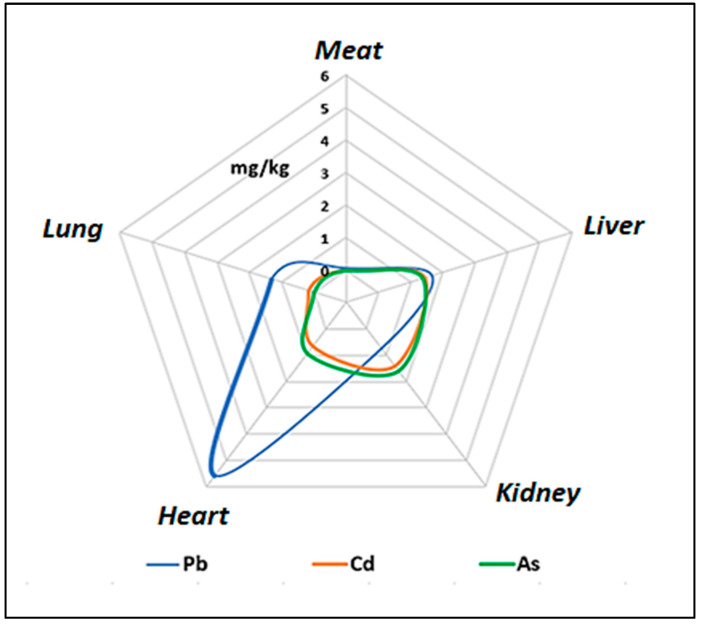
Radial graph of Pb, Cd, and As content in different meat and viscera of guinea pigs. Pb accumulation is significantly higher in the heart compared to other tissues, while Cd and As levels are similar and more homogeneous, with higher concentrations in the liver and kidney. These results reflect differences in heavy metal bioaccumulation depending on the function and metabolism of each tissue.

The average amount of Pb, Cd, and As in meat, kidneys, lungs, heart, and liver, in order of importance, was Pb > Cd > As. The average concentrations of these toxic metals and metalloids in meat produced in the study areas were above the maximum permissible limits regulated internationally (Figure 2).

The radial graph visually shows that the highest concentration of Pb is observed in the heart, with lower concentrations in the liver and kidneys and even lower in the meat, while the contents of Cd and As were similar in the heart, liver, and kidneys and were not detected in the meat or the lungs.

### 3.6. Potential Health Risks

**Figure 3:**
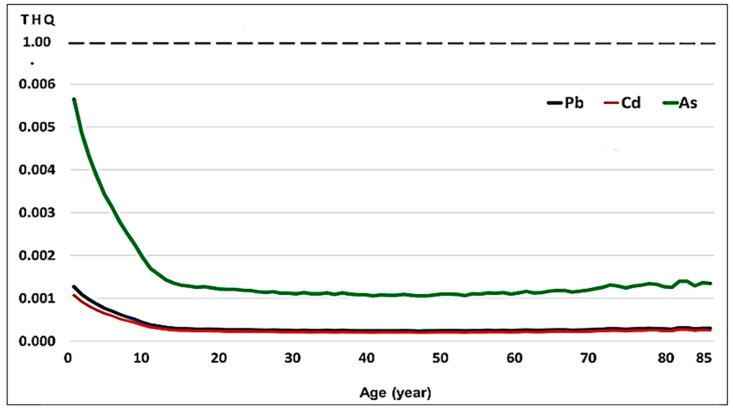
Curves of the Target Hazard Quotient (THQ) for Pb, Cd, and As for consumption of guinea pig produced in the Central Andes in the Peruvian population aged 2–85 years.

The THQs for Pb, Cd, and As due to guinea pig consumption were below the value of 1 at all ages, with no risk whatsoever from the consumption of guinea pig produced in the Central Andes of Peru (Table 7, Table 8 and Table 9, and Figure 3). On average, the THQ for As was 5.3 times higher than the THQ for Pb and Cd, indicating that this metalloid would be the main contaminant of guinea pig meat and offal under the breeding conditions at the study site. It is also observed that the THQs for Pb, Cd, and As are higher the younger the people are. From approximately 15 years of age onwards, the values become lower and remain similar until 85 years of age.

**Figure 4:**
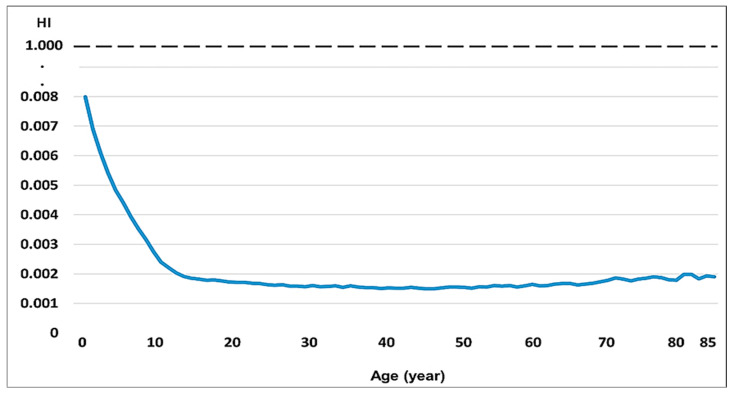
Hazard Index (HI) curve for the Peruvian population aged 3–85 years for consumption of guinea pig meat produced in the Central Andes.

The Hazard Index (HI) was below 1, not representing a risk for its consumption (Table 10, Figure 4).

The average cancer risk values associated with guinea pig consumption for Pb, Cd, and As were 4.47 × 10^−8^, 3.55 × 10^−5^, and 3.87 × 10^−6^ for the study population. The total cancer risk for the Peruvian population was 3.94 × 10^−5^, a value that did not exceed the acceptable limit of 1 × 10^−6^ to 1 × 10^−4^ [45].

## 4. Discussion

### 4.1. Edible Components of the Guinea Pig Carcass

The guinea pig, in its different gastronomic presentations, includes not only the meat or muscle fraction, skin, and accumulated fat, but also the liver, kidneys, heart, and lungs. In an average 800 g carcass, approximately 750 g are meat, skin, and fat, and the rest of the organs or offal weigh approximately 50 g. These values are within the range determined in other works. An FAO publication reported that the average weights of the liver, kidneys, heart, and lungs were 23.29 ± 6.03, 6.06 ± 1.43, 2.79 ± 0.76, and 4.85 ± 1.51 g, respectively [46]. In another study, the weight of the liver is reported to be 20.3 g [47]. In another study, in guinea pigs slaughtered at 72 days of age and fed a mixed diet (80% ryegrass, plus 20% concentrate), the relative weights of the liver, lungs, heart, and kidneys were reported to be from 3.41 to 4.41, 0.57 to 0.78, 0.30 to 0.41, and 1.07 to 1.20%, respectively [48]. If the weight of each organ in an 800 g carcass is calculated, the liver, lungs, heart, and kidneys would have average weights ranging from 27.28 to 35.28, 4.56 to 6.24, 2.4 to 3.26, and 8.56 to 9.60 g, respectively, and our results are between these ranges.

### 4.2. Lead, Cadmium, and Arsenic Levels in Guinea Pig Meat, Liver, Kidney, Heart, and Lungs

Pb, Cd, and As concentrations differed among the different edible parts of the guinea pig, being lower in the meat than in the viscera. All samples had concentrations below the LMP established by the USDA [28]. The highest concentrations of Pb were recorded in the heart, Cd in the liver and kidneys, and As in the kidneys, while the meat had the lowest contents in the following order: Pb > Cd > As. Even though the levels of Pb, Cd, and As were low, the heart is found to have more than 11 times more Pb than the MPL (Figure 1). The Pb concentrations in descending order were as follows: heart > liver > lungs > kidneys > meat (muscle and skin). These results support other studies that point to the liver and kidneys as organs with a high Pb load; however, these studies do not report contents in the heart.

Although the largest proportion of absorbed Pb is deposited in the skeleton, which contains more than 90% of the body load of Pb [49], it is observed that, among soft tissues, the liver and kidney reach the highest concentrations, but it is also found in most body tissues, including muscle [50,51]. Blood, soft tissues, and bones are therefore the main kinetic reservoirs of the body burden of Pb [52].

In sheep reared in a former mining area of the Sierra Madrona and Alcudia Valley (Spain), blood samples were taken before slaughter, and liver and muscle samples were taken. They reported that blood, liver, and muscle Pb levels were higher in the mining area than in the low contamination control area. The blood Pb concentration in the mining area was 6.7 μg/dL in sheep and 10.9 μg/dL in rams, which are values above background levels (>6 μg/dL) in 73.3% of the animals. The concentration of Pb in the liver of 68% of the sheep from the mining area was 6.16 μg/g dry weight (dw) and exceeded the minimum level associated with toxic exposure (5 μg/g dw), and 87.5% of the liver samples, were above the EU MRLs established for offal intended for human consumption (0.5 µg/g dw~1.4 µg/g dw). In contrast, none of the sheep muscle samples exceeded the EU MPL (0.1 µg/g wet weight ~0.34 µg/g dry weight) established for meat. These results suggest a possible effect on the health of sheep exposed to Pb contamination in this area and possible implications for food safety for human consumers of meat and offal from these animals [53].

In chicken meat, the authors of [54] reported a Pb and Cd content of 0.03 ± 0.006 and 0.008 ± 0.002 mg/kg, and they reported values of 0.03 ± 0.013 and 0.058 ± 0.026 mg/kg for Pb and Cd in the liver; also, for gizzard, they reported values of 0.019 ± 0.009 (Pb) and 0.018 ± 0.008 (Cd), indicating that the maximum permissible values for meats is 0.1 and 0.5 mg/kg for Pb and Cd, respectively [55,56]. In red meat, the contents of As, Pb, and Cd were reported as 24.35, 480.86, and 171.134 µg/kg, with the MPL for As, Pb, and Cd being 500, 100, and 50 µg/kg [57]. In trout muscle, the Pb content in the continuous flow system was reported as 0.044 ± 0.02 mg/kg; for the recirculation system, it was 0.061 ± 0.02 mg/kg, and for Cd, in the same systems, it was 0.011 ± 0.002 mg/kg and 0.013 ± 0.001 mg/kg, respectively, indicating that the MPL according to the EU Regulation 2023/915 for Pb and Cd is 0.3 and 0.5 mg/kg, respectively [58]. Therefore, the consumption of trout meat from the studied farms did not pose a significant threat to consumer health. In another study that evaluated the presence of toxic and essential metals in the liver, kidney, and muscle of pigs from a slaughterhouse in Galicia, northwest Spain, using ICP-OES and ICP-MS, they reported that the average concentrations of Cd in the liver, kidney, and muscle were 0.073, 0.308, and 0.009 mg/kg; 0.004, 0.008 and 0.003 mg/kg for Pb; and 0.013, 0.011, and 0.003 mg/kg for As. These concentrations can be considered low, and, in general, similar to those reported in other studies in Spain. None of the samples exceeded the maximum admissible concentrations established by the European Union, so they are safe for consumption [59].

In cuts of loin and neck obtained from Creole cattle in various municipalities of Antioquia, the authors of [60] reported average values of Pb at 0.041 ± 0.016 and, for Cd, values < 0.010 mg/kg; the levels were within the values allowed by Colombian regulations for all cases, which establish limits of 0.050 mg/kg and 0.10 mg/kg for Cd and Pb, coinciding with the limits established by the European Union for meat products Reg. EU. 37/2010 [61].

In Baquba, Diyala province, Iraq, using atomic absorption spectroscopy (AAS), the concentrations of Cd and Pb in the heart, kidneys, and meat of beef (cow), lamb (sheep), and chicken were determined, among other metals. Heavy metal levels in beef, lamb, chicken, heart, kidney, and meat ranged from 0.058 to 0.680 ppm Cd and 2.269 to 5.726 ppm Pb [62]. Overall, meat was found to have the highest and significant levels of metals, and heart and kidney had the lowest. Significant differences in heavy metal concentrations were also observed in beef, lamb, and chicken. Pb and Cd concentrations exceeded the permissible limits set by WHO/FAO [63].

In a study conducted in Fars province (south of Iran), Pb and Cd contents in muscle, liver, and kidney samples of cattle and their relationship with their concentrations in the feed consumed were reported. The arithmetic means concentrations (mg/kg wet weight) of Pb and Cd were 0.221 and 0.028 in the muscle, 0.273 and 0.047 in the liver, and 0.244 and 0.114 in the kidney. All measured concentrations (with the exception of Pb in muscle) were below the EU MPLs. Cd contents in kidneys were significantly higher than those observed in other tissues [64].

Our findings show that each element accumulates differently in meat and offal and is affected by the level of emissions from anthropogenic activities in adjacent areas. However, no risk of toxicity from Pb, Cd, and As is expected due to human consumption of guinea pigs due to the low per capita consumption of this species.

### 4.3. Health Risk in the Peruvian Population Aged 3–85 Years Due to Consumption of Guinea Pig Meat

The health risk assessment associated with Pb, Cd, and As contamination, in the edible portion of guinea pig, was calculated by estimating the Target Hazard Quotient (THQ), the Hazard Index (HI), and the cancer risk (CR). For this, 50 samples of meat and offal (meat, heart, liver, kidneys, and lungs) were used. The health protection standard for lifetime risk for THQ and HI is 1.0 [65], and for cancer risk, values from 1 × 10^−6^ to 1 × 10^−4^ are considered acceptable or tolerable [45].

In the present study, the THQ and HI values of Pb, Cd, and As concentrations from guinea pig consumption in the Peruvian population aged 2–85 years (Figure 2) were below the safe limit (1.0). Consequently, our findings demonstrated that the intake of these metals from guinea pig consumption is safe and will not cause carcinogenic risks to consumers. The THQ values for Pb, Cd, and As and the HI for these elements were below unity, such that the guinea pig meat and offal that are part of the consumable portions were found to be well below 1 (Figure 3). However, considering that the per capita consumption of guinea pigs is only 660 g/year, even when more than 5 kg are consumed per year, the HI is well below the value of 1, which indicates little risk to consumers; thus, the consumption of this nutritious meat should be promoted and contributes to the nutritional food security of the Andean populations.

We evaluated the THQ for Pb, Cd, and As based on guinea pig consumption in the Peruvian population. The THQ of exposure to these elements with age showed an inverse relationship, with younger children having the highest intake of toxic metals and metalloids from guinea pig milk consumption and the lowest body weight, therefore presenting the highest TQH value. This suggests that guinea pig meat and offal consumed could be an important source of toxic metals and metalloids for young children. The exposure to Pb, Cd, and As for children aged 2, 3, and 5 years is 4.78, 4.12, 3.64, and 3.24 times that of an adult aged 25 years and weighing 65 kg, i.e., based on body weight [66].

THQ values followed a descending order of As > Pb > Cd. A previous study conducted on milk in the Central Andes of Peru showed that the THQ followed a descending order of As > Pb > Cd, with values of 0.05–1.13, 0.01–0.28, and 0.01–0.24 for the minimum milk intake in people aged 2–85 years; 0.09–1.41, 0.02–0.35, and 0.02–0.30 for the average intake; and 0.13–1.69, 0.03–0.42, and 0.03–0.36 for the maximum milk intake, with higher values at younger ages [67]. Other studies, also on fresh cow milk, showed that As had the highest THQ value [68,69].

The HI values for guinea pig consumption in children aged 2, 3, 4, and 5 years were 4.9, 4.1, 3.6, and 3.2 times higher than for a 25-year-old adult weighing 65 kg. For the population aged 2–85 years, the HI values were between 0.0019 and 0.0080, well below the threshold of 1. This result indicates that these levels of human exposure to Pb, Cd, and As would not have any adverse effects during a consumer’s lifetime. However, it should be noted that only guinea pig consumption was considered in this study because the varied daily diet produced under the conditions of the study area could increase substantially. Guinea pig consumption makes a low contribution to total dietary toxic metal and metalloid intake among local residents. For example, milk consumption contributes significantly to the intake of these elements [70,71].

In a study on dietary exposure to heavy metals in raw milk in the vicinity of leather processing plants in China, they report HI values between 0.0124 and 0.0832, well below the THQ threshold of 1 for individuals aged 3 to 69 years, indicating no risk to consumers; however, in our study, milk could make a low contribution to total dietary heavy metal intake in the studied region [72].

In the USA, considering their toxicity and frequency of occurrence, As, Pb, and Cd have been established as the first, second, and seventh priority food contaminants [73]; therefore, we recommend the establishment of monitoring and risk assessment programs for heavy metal concentrations in foods produced in the Central Andes, where mining and metallurgical activities contaminate different foods produced under the environmental conditions of this region of the world. The results could be used to manage and establish regulations that reduce human exposure to these metals.

### 4.4. Implication of Pb, Cd, and As Intake on Health

The accumulation of heavy metals, such as lead (Pb), cadmium (Cd), and arsenic (As), in the soils and grasslands of the Central Andes of Peru, which is exacerbated by mining/metallurgical activities, the use of phosphate fertilizers, and irrigation with contaminated water, represents a significant threat to public and mental health. These sources of contamination introduce toxic metals and metalloids into the food chain, increasing the risks associated with their human consumption. Pb, even at low levels, is associated with significant neurotoxic effects, particularly in vulnerable populations such as children and pregnant women [74,75].

Previous studies in Peru, such as those carried out in communities near the Las Bambas mining project, have shown a negative impact on child psychomotor development, with a 12.5% risk in children under three years of age according to the TEPSI test, and alterations in the IQ assessed using the Stanford–Binet test [76]. At a global level, research indicates that joint exposure to Pb, Cd, and As contributes to cognitive impairment, depressive disorders, and anxiety disorders, including generalized anxiety, agoraphobia, and social anxiety disorder [77,78,79].

In adults, exposure to Pb, Cd, and mercury (Hg) is correlated with adverse effects on neurocognitive function, including impairments in memory, attention, and executive functions. These neurotoxic effects are linked to mechanisms such as oxidative stress, neurotransmitter disruption, and metal accumulation in brain tissue, affecting critical regions such as the hippocampus and prefrontal cortex [80,81]. These findings underscore the urgency of public policies that reduce exposure to these metals, especially during pregnancy and lactation, stages in which neurological effects can be more severe and permanent.

Scientific evidence highlights that mitigating these exposures can prevent neurodevelopmental delays and improve the quality of life of affected communities. Likewise, empirical research needs to be strengthened to validate and expand these findings, particularly in contexts such as the Central Andes, where the interaction between environmental factors and social vulnerability is critical to understanding the impact of toxic metals and metalloids on public health [71,82,83].

The implementation of environmental monitoring strategies, health education, and strict regulation of industrial activities is essential to protect at-risk populations and address this problem with a sustainable and inclusive approach.

## 5. Conclusions

In this study, the levels of As, Pb, and Cd in guinea pig meat and offal in farms in the Central Andes of Peru did not exceed the internationally established limit values. The higher concentrations of As and Pb in guinea pig meat and offal in the study area could be due to the use of contaminated irrigation water for pastures and phosphate rock fertilizers, which are the main sources of Cd. These elements pass from the soil to the pasture and guinea pigs; however, due to the low per capita consumption of guinea pigs in the Peruvian population, the TQH and HI were below 1. Our risk assessment suggested that the concentrations of As, Pb, and Cd in guinea pig meat and offal that are part of the guinea pig consumption portions do not cause health risks to the Peruvian population between 2 and 85 years of age. The findings of this study expand the scientific basis for the safe and harmless production of guinea pigs in the Central Andes of Peru and are important for developing guidelines and standards that ensure that meat products are safe and harmless for human consumption. We urge the establishment of standards and maximum limits for heavy metals and arsenic in water, soil, pastures, and products produced in the Central Andes.

## Figures and Tables

**Table 1:** Operating conditions of the MP-AES (Agilent-4200) for the determination of Pb, Cd, and As in guinea pig meat and offal.

Element	Wavelength (nm)	Nebulizer Flow (L/min)	Replicas	Pump Speed (rpm)	Reading Time (s)
Pb	220.353	15.0	3	15	3
Cd	226.502	15.0	3	15	3
As	228.812	15.0	3	15	3

**Table 2:** Maximum limits of Pb, Cd, and As in meat and meat products, including edible viscera (mg/kg).

Meats and Meat Products	Pb	Cd	As
Meats (excluding organ meats from livestock and poultry and their products)	0.2	0.1	0.5
Organ meats from livestock and poultry	0.5	-	0.5
Meat products (excluding organ meats from livestock and poultry)	0.3	0.1	0.5
Liver from livestock and poultry	0.5	0.5	0.5
Kidney from livestock and poultry	0.5	1.0	0.5

Source: Lead limits in foods [28].

**Table 3:** Components of an edible portion of a guinea pig carcass.

Edible Component	Grams	%
Meat	749.5	93.69
Liver	30.86	3.86
Kidneys	10.47	1.31
Heart	4.85	0.61
Lungs	4.32	0.54
Total	800	100.00

**Table 4:** Concentration of lead in guinea pig meat and offal (mg/kg).

Samples	*n*	Mean	Standard Deviation	95% Confidence Interval for the Mean		Min	Max
				Lower Limit	Upper Limit		
Meat	10	0.069 b	0.060	0.025	0.112	<LOD	0.183
Liver	10	1.669 b	0.355	1.415	1.923	1.232	2.356
Kidneys	10	0.765 b	0.289	0.559	0.972	0.294	1.183
Heart	10	5.583 a	3.593	3.013	8.153	1.249	11.946
Lungs	10	1.312 b	0.684	0.823	1.802	0.341	2.010

a, b, Mean values per sample with different letters vary statistically (*p* < 0.05). Each animal generated a sample of meat, liver, kidneys, heart, and lungs. Thus, the 10 animals generated a total of 50 samples.

**Table 5:** Concentration of cadmium in guinea pig meat and offal (mg/kg).

Samples	*n*	Mean	Standard Deviation	95% Confidence Interval for the Mean		Min	Max
				Lower Limit	Upper Limit		
Meat	10	0.002 b	0.006	0.002	0.007	<LOD	0.020
Liver	10	1.445 a	0.730	0.923	1.967	0.684	2.745
Kidneys	10	1.420 a	1.002	0.703	2.137	<LOD	3.096
Heart	10	0.652 ab	1.960	0.749	2.055	<LOD	6.227
Lungs	10	0.158 ab	0.282	0.044	0.359	<LOD	0.941

a, b, Mean values per sample with different letters vary statistically (*p* < 0.05).

**Table 6:** Arsenic concentration in guinea pig meat and offal (mg/kg).

Samples	*n*	Mean	Standard Deviation	95% Confidence Interval for the Mean		Min	Max
				Lower Limit	Upper Limit		
Meat	10	<LOD	<LOD	<LOD	<LOD	<LOD	<LOD
Liver	10	1.385 ab	0.574	0.975	1.795	0.775	2.409
Kidneys	10	1.607 a	1.231	0.726	2.487	<LOD	3.358
Heart	10	0.934 ab	2.207	0.645	2.513	<LOD	6.783
Lungs	10	<LOD	<LOD	<LOD	<LOD	<LOD	-

a, b, Mean values per sample with different letters vary statistically (*p* < 0.05).

**Table 7:** Target Hazard Quotient (THQ) for lead due to guinea pig consumption in the Peruvian population aged 2–85 years.

Age	THQ-Pb	Age	THQ-Pb	Age	THQ-Pb	Age	THQ-Pb
2	0.0013	25	0.0003	48	0.0002	71	0.0003
3	0.0011	26	0.0003	49	0.0002	72	0.0003
4	0.0010	27	0.0003	50	0.0002	73	0.0003
5	0.0009	28	0.0003	51	0.0002	74	0.0003
6	0.0008	29	0.0003	52	0.0002	75	0.0003
7	0.0007	30	0.0003	53	0.0002	76	0.0003
8	0.0006	31	0.0002	54	0.0002	77	0.0003
9	0.0006	32	0.0003	55	0.0002	78	0.0003
10	0.0005	33	0.0002	56	0.0003	79	0.0003
11	0.0004	34	0.0002	57	0.0003	80	0.0003
12	0.0004	35	0.0003	58	0.0003	81	0.0003
13	0.0004	36	0.0002	59	0.0002	82	0.0003
14	0.0003	37	0.0003	60	0.0003	83	0.0003
15	0.0003	38	0.0002	61	0.0003	84	0.0003
16	0.0003	39	0.0002	62	0.0003	85	0.0003
17	0.0003	40	0.0002	63	0.0003		
18	0.0003	41	0.0002	64	0.0003		
19	0.0003	42	0.0002	65	0.0003		
20	0.0003	43	0.0002	66	0.0003		
21	0.0003	44	0.0002	67	0.0003		
22	0.0003	45	0.0002	68	0.0003		
23	0.0003	46	0.0002	69	0.0003		
24	0.0003	47	0.0002	70	0.0003		

THQ: Target Hazard Quotient. A value > 1 indicates a potential risk of having effects on health [44].

**Table 8:** Target Hazard Quotient (THQ) for cadmium due to guinea pig consumption in the Peruvian population aged 2–85 years.

Age	THQ-Cd	Age	THQ-Cd	Age	THQ-Cd	Age	THQ-Cd
2	0.0011	25	0.0002	48	0.0002	71	0.0002
3	0.0009	26	0.0002	49	0.0002	72	0.0002
4	0.0008	27	0.0002	50	0.0002	73	0.0002
5	0.0007	28	0.0002	51	0.0002	74	0.0002
6	0.0006	29	0.0002	52	0.0002	75	0.0002
7	0.0006	30	0.0002	53	0.0002	76	0.0002
8	0.0005	31	0.0002	54	0.0002	77	0.0003
9	0.0005	32	0.0002	55	0.0002	78	0.0003
10	0.0004	33	0.0002	56	0.0002	79	0.0002
11	0.0004	34	0.0002	57	0.0002	80	0.0002
12	0.0003	35	0.0002	58	0.0002	81	0.0003
13	0.0003	36	0.0002	59	0.0002	82	0.0003
14	0.0003	37	0.0002	60	0.0002	83	0.0002
15	0.0003	38	0.0002	61	0.0002	84	0.0003
16	0.0002	39	0.0002	62	0.0002	85	0.0003
17	0.0002	40	0.0002	63	0.0002		
18	0.0002	41	0.0002	64	0.0002		
19	0.0002	42	0.0002	65	0.0002		
20	0.0002	43	0.0002	66	0.0002		
21	0.0002	44	0.0002	67	0.0002		
22	0.0002	45	0.0002	68	0.0002		
23	0.0002	46	0.0002	69	0.0002		
24	0.0002	47	0.0002	70	0.0002		

THQ: Target Hazard Quotient. A value > 1 indicates a potential risk of having health effects [44].

**Table 9:** Target Hazard Quotient (THQ) for arsenic from guinea pig consumption in the Peruvian population aged 2–85 years.

Age	THQ-As	Age	THQ-As	Age	THQ-As	Age	THQ-As
2	0.0056	25	0.0012	48	0.0011	71	0.0013
3	0.0049	26	0.0012	49	0.0011	72	0.0013
4	0.0043	27	0.0011	50	0.0011	73	0.0013
5	0.0038	28	0.0012	51	0.0011	74	0.0012
6	0.0034	29	0.0011	52	0.0011	75	0.0013
7	0.0031	30	0.0011	53	0.0011	76	0.0013
8	0.0028	31	0.0011	54	0.0011	77	0.0013
9	0.0025	32	0.0011	55	0.0011	78	0.0013
10	0.0022	33	0.0011	56	0.0011	79	0.0013
11	0.0019	34	0.0011	57	0.0011	80	0.0013
12	0.0017	35	0.0011	58	0.0011	81	0.0014
13	0.0016	36	0.0011	59	0.0011	82	0.0014
14	0.0014	37	0.0011	60	0.0011	83	0.0013
15	0.0014	38	0.0011	61	0.0012	84	0.0014
16	0.0013	39	0.0011	62	0.0011	85	0.0013
17	0.0013	40	0.0011	63	0.0011		
18	0.0013	41	0.0011	64	0.0012		
19	0.0013	42	0.0011	65	0.0012		
20	0.0012	43	0.0011	66	0.0012		
21	0.0012	44	0.0011	67	0.0011		
22	0.0012	45	0.0011	68	0.0012		
23	0.0012	46	0.0011	69	0.0012		
24	0.0012	47	0.0011	70	0.0012		

THQ: Target Hazard Quotient. A value > 1 indicates a potential risk of having health effects [44].

**Table 10:** Hazard Index (HI) in the Peruvian population aged 3–85 years for consumption of guinea pig meat produced in the Central Andes of Peru.

Age	HI	Age	HI	Age	Hi	Age	HI
2	0.0080	25	0.0017	48	0.0015	71	0.0018
3	0.0069	26	0.0016	49	0.0015	72	0.0019
4	0.0061	27	0.0016	50	0.0016	73	0.0018
5	0.0054	28	0.0016	51	0.0016	74	0.0018
6	0.0048	29	0.0016	52	0.0015	75	0.0018
7	0.0044	30	0.0016	53	0.0015	76	0.0019
8	0.0039	31	0.0016	54	0.0016	77	0.0019
9	0.0035	32	0.0016	55	0.0016	78	0.0019
10	0.0032	33	0.0016	56	0.0016	79	0.0018
11	0.0027	34	0.0016	57	0.0016	80	0.0018
12	0.0024	35	0.0016	58	0.0016	81	0.0020
13	0.0022	36	0.0015	59	0.0016	82	0.0020
14	0.0020	37	0.0016	60	0.0016	83	0.0018
15	0.0019	38	0.0016	61	0.0016	84	0.0019
16	0.0019	39	0.0015	62	0.0016	85	0.0019
17	0.0018	40	0.0015	63	0.0016		
18	0.0018	41	0.0015	64	0.0017		
19	0.0018	42	0.0015	65	0.0017		
20	0.0018	43	0.0015	66	0.0017		
21	0.0017	44	0.0015	67	0.0016		
22	0.0017	45	0.0015	68	0.0017		
23	0.0017	46	0.0015	69	0.0017		
24	0.0017	47	0.0015	70	0.0017		

HI: Hazard Index: A value > 1 indicates a potential risk of having health effects [44].

## Data Availability

Data are contained within the article and Supplementary Materials.

## Supplementary Materials

The following supporting information can be downloaded at: https://www.mdpi.com/article/10.3390/vetsci12040292/s1.
